# Piplartine attenuates the proliferation of hepatocellular carcinoma cells via regulating hsa_circ_100338 expression

**DOI:** 10.1002/cam4.3043

**Published:** 2020-04-13

**Authors:** Xiaoli Cheng, Pan Tian, Wengzhong Zheng, Xuetao Yan

**Affiliations:** ^1^ Department of Pharmacy Bao'an Maternal and Child Health Hospital Jinan University Shenzhen China; ^2^ Department of Anesthesiology Bao'an Maternal and Child Health Hospital Jinan University Shenzhen China

**Keywords:** competing endogenous RNAs, Hepatocellular carcinoma, hsa_circRNA_100338, piplartine, proliferation

## Abstract

Researches have pointed that piplartine inhibits the proliferation of hepatocellular carcinoma (HCC) cells, however, the underlying mechanisms has not been well defined. Currently, more and more studies have pointed out that circRNAs can regulate tumor cell proliferation, involve in the tumorigenesis mechanism of various tumors. In this study, we explored whether piplartine may participate in the development of HCC through the regulation of ability of HCC cell proliferation by circRNA. Based on the chip analysis, we selected candidate circRNAs that are highly correlated with HCC. CircRNA expression in OSCC cells treated with piplartine was detected by qRT‐PCR. We found that only the expression of hsa_circ_100338 (circ‐100338) was observably reduced. The expression characteristics of circ‐100338 in HCC cell lines were also verified by qRT‐PCR. Subsequently, whether or notcirc‐100338 can regulate ZEB1 via competitively binding to miR‐141‐3p was determined by the RIP assay and dual luciferase reporter gene assay. The effect of the circ‐100338/miR‐141‐3p/ZEB1 axis on the proliferation of HCC cell was tested by EdU and CCK‐8 assay. Results showed that circ‐100338 expression was observably increased in HCC cell lines. Simultaneously, circ‐100338 can regulate the expression of ZEB1by competitively binding to miR‐141‐3p. Moreover high expression of circ‐100338 can stimulate the proliferation of HCC cells. Our current study revealed that circ‐100338 played as a ceRNA in promoting the progression of HCC by sponging miR‐141‐3p, while piplartine can participate in the development of HCC by inhibiting the expression of circ‐100338.

## Graphical abstract

Hsa_circ_100338 was upregulated in piplartine treated hepatocellular carcinoma cells. Dual luciferase reporter gene assay and RIP assay showed that circ‐100338 can regulate ZEB1 expression by competitively bind to miR‐141‐3p.

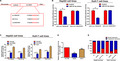

## 1 INTRODUCTION

Hepatocellular carcinoma (HCC) is the commonest liver cancer^1^ and is one of leading cause of cancer‐related mortality. According to reports, more than 600 000 people die of HCC each year.^2^ The majority of HCC occurs in sub‐Saharan Africa and Asia, and it is more common in men rather than women.^3^ Epidemiological and experimental studies have pointed out that the development of HCC may be caused and promoted by hepatitis B virus (HBV) and hepatitis C virus infection (HCV),^4^ smoking, alcohol,^5^ etc In recent years, many people have studied HCC from a genetic perspective and have achieved some results. For example, Salpini R et al demonstrated the novel HBx mutation F30V reduces HBV replication ability, increases the anti‐apoptotic ability of HBx N‐terminus in vitro and associates with HCC in vivo.^6^ Zhang et al confirmed reducing of exosome transmitted miR‐320a from cancer‐associated fibroblasts promotes HCC metastasis and proliferation.^7^ But these findings have not yet fully revealed the pathogenesis of HCC, thereby it has important clinical value to further research the occurrence and development of HCC from the epigenetic perspective.

CircRNA is a non‐protein‐encoding RNA^8^ that is generated from back splicing and is widely found in eukaryotic genomes.^9^ Its structure is special, closed loop shape,^10^ rich in microRNA (miRNA) binding sites, and acts as a miRNA sponge in cells.^11^ CircRNA is a hot topic in recent years, so there are more research results. CircRNA is associated with many diseases, including nonalcoholic steatohepatitis, human cartilage degenerative of osteoarthritis, Alzheimer's disease, osteosarcoma,^12^, ^13^, ^14^, ^15^ etc Studies by Huang XY et al confirmed that circRNA can also participate in the progression of HCC.^16^

Piperlongumine (piplartine) is a natural alkaloid isolated from pepper. Piplartine can selectively induce reactive oxygen species production and increase death of cancer cells.^17^ Piplartine is one of the nuclear export inhibitors with potential anticancer activity.^18^ Studies showed that in breast cancer cells piplartine can downregulate the expression of HER family.^19^ Karki K et al found that lung cancer cell lines treated with piplartine can inhibit Cell proliferate and induce apoptosis.^20^ Indeed, previous studies have confirmed that piplartine has a therapeutic effect on hepatocellular carcinoma.^21^ But whether it can affect the regulation of expression of circRNA to participate in tumorigenesis mechanism requires further exploration, so we have made a further study from the perspective of epigenetics.

In conclusion, we first assessed the effect of piplartine on the circRNA expression in HCC. We confirmed that the piplartine suppresses proliferation of hepatocellular carcinoma by circ‐100338 function as competitive endogenous RNA by a sequence of assays.

## 2 MATERIALS AND METHODS

### 2.1 Cell culture and transfection

All cells were purchased from Shanghai Institutes for Biological Science, cultured with RPMI 1640 complete medium (Hyclone), and grown in a carbon dioxide incubator at 37°C with 5% of carbon dioxide.

### 2.2 RNA isolation and real‐time quantitative PCR (qRT‐PCR) detection

Total RNAs were extracted and reversely transcribed into cDNA using TRIzol reagent (Beyotime). Then Power SYBR Green (Takara) was used to perform qRT‐PCR. GAPDH expression was used to normalize Data. PCR primers were listed in Table S1, and the 2^−ΔΔCt^ was used to calculate the relative levels.

### 2.3 RNase R digestion

Total RNAs were incubated 20 minutes with RNase R (Epicentre Biotechnologies) at 37°C. The RNase R digestion reactions were carried out according to previously published procedures.

### 2.4 Dual‐luciferase reporter assay

The 3′‐UTR sequence of circ‐100338 and ZEB1 containing the binding site of miR‐141‐3p was cloned into the KpnI and SacI sites of pGL3 promoter vector (Realgene). HCC cells cultured in 24‐well plates were co‐transfected with plasmid including 80 ng plasmid, 5 ng renilla luciferase vector pRL‐SV40, 50 nmol/L miR‐141‐3p mimics and its negative control using lipofectamine 2000 (Invitrogen). After 24 hours of transfection, the relative activity of luciferase was normalized by the ratio of the Firefly luminescence to the Renilla luminescence examined by Dual‐Luciferase Reporter Assay following the manufacturer's instructions.

### 2.5 Subcellular fractionation location

According to the protocol, nuclear and cytoplasmic RNAs were separated using PARIS Kit (Life Technologies). As mentioned above, total RNA of each component was determined by qRT‐PCR. GAPDH was the cytoplasmic marker and U6 was the nuclear control transcript.

### 2.6 Cell proliferation

Cell multiplication was performed using the cell count kit‐8 (CCK8) assay (Promega) every 24 hours according to the manufacturer instruction. In short, the cells after transfection were plated in 96‐well plates at 3000 cells/well, and then incubated with CCK8 solution for 2 hours. Cell viability was measured spectrophotometrically at 450 nm.Cell proliferation was detected by 5‐acetylene 2‐deoxyuridine (EdU)kit (Ribobio). Cells were plated in 96‐well plates at 5 × 10^3^ cells/well.EdU labeling media was added into the 96‐well plates 48 hours after transfection, and were incubated for 2 hours under 5% of carbon dioxide at 37°C. After treatment of cells with 4% paraformaldehyde and 0.5% Triton X‐100, cells were stained with anti‐EdU working solution. The nucleus was labeled by DAPI. The percentage of EdU positive cells in vitro culture was calculated by fluorescence microscopy. Each treatment group was calculated by randomly selecting for five fields of view.

### 2.7 Animal experiments

We purchased BALB/c thymic‐free nude mice (female, 4‐5 weeks old) from Animal center of Peking University. HuH‐7 cells were subcutaneously injected into the flanks of mice. Four weeks after cell injection, mice were sacrificed and tumors were resected for further analysis. The study got the approval of the ethics committee in Bao'an Maternal and Child Health Hospital, Jinan University, and the animal experiments were performed according to the NIH requirement.

### 2.8 IHC staining analysis

Expression of Ki‐67in hepatocellular carcinoma tissue in vivo was analyzed by immunohistochemical staining method using tumor tissue embedding paraffin section burdened specimens from nude mice. After fixing with 10% neutral formalin, samples were then embedded in paraffin after dehydration. The paraffin embedded tissue was cut into 5 µm thick sections. The sections were subjected to conventional dewaxing, antigen microwave heat recovery and inactivation of endogenous peroxidase. The sections were incubated with anti‐Ki‐67 primary antibody (Abcam) overnight at 4°C. Subsequently, the sections were incubated with secondary antibody at room temperature for 30 minutes. After incubation for an additional 30 minutes at room temperature with a polymerase adjuvant, all the sections were stained with 3,3'‐diaminobenzidine. After counterstaining with 0.02% hematoxylin, the sections were then observed by a microscope, and the photoshop was used to process the images.

### 2.9 Western blot detection

The total protein was extracted from the cultured cells with RIPA lysate (Beyotime). Take 10 μL of at least 40 μg of protein for each gel hole of upper 4% stacking gel, and GAPDH expression was used as an internal reference to ensure consistent sample loading. Image J software (NIH) was used for semi‐quantitative grayscale analysis of each target protein bands.

### 2.10 Statistical analysis

All in vitro experiments are repeated at least three times in different time periods, and the results are expressed as mean plus or minus standard deviation. SPSS 22.0(NDTimes) was used to statistically analyze all the above experimental results with One‐way ANOVA test or Student's *t* test. *P* < .05 is considered to be statistical significance.

## 3 RESULTS

### 3.1 Characteristics of circ‐100338 in HCC

**Figure 1:**
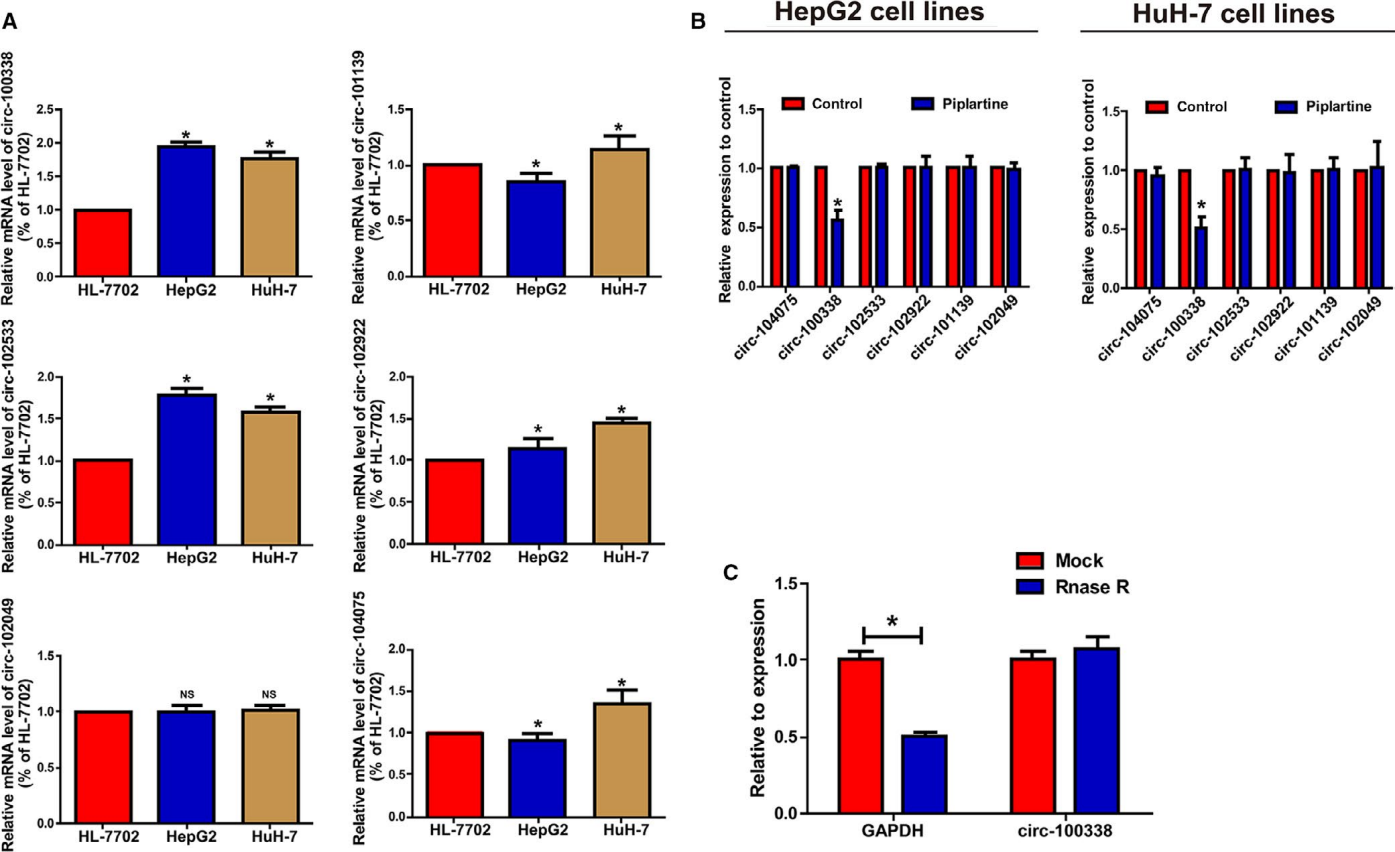
Characteristics and expression of circ‐100338 in HCC. A, We examined the expression levels of six candidate circRNAs by qRT‐PCR in HepG2 and HuH‐7 of HCC cell lines and human normal liver cell line HL‐7702, the expression of circ‐100338 and circRNA‐102533 were significantly elevated in HCC cell lines compared to HL‐7702 cell line. B, After treatment of HepG2 and HuH‐7 cell line with piplartine, the expression level of circ‐100338 was decreased. C, Circ‐100338 has significant resistance to RNaseR digestion

The microarray results of Huang et al demonstrated that six circRNAs expression in HCC tissues were significant different from that in adjacent control tissues, and the six circRNAs include namely hsa_circRNA_100338, hsa_circRNA_102922, hsa_circRNA_104075, hsa_circRNA_101139, hsa_circRNA_102049, and hsa_circRNA_102533.^16^ To further explore the levels of the top six circRNAs in HCC cell lines, we selected human normal liver cell line HL‐7702 as control. The results demonstrated that the level of circ‐100338 and circRNA‐102533 in HCC cell lines was significantly increased compared toHL‐7702(Figure 1A), which is consistent with the chip prediction. At the same time, we treated the HCC cell lines with piplartine, and then detected the expression of these top six circRNAs in HCC cell lines by qRT‐PCR. The results demonstrated that only the expression of circ‐100338 was lowered obviously after treatment of HCC cell lines with piplartine (Figure 1B). These results indicate that piplartine may be related to the progression of HCC by influencing the expression of circ‐100338. Meanwhile, in order to further verify circ‐100338’s circular nature, we designed the experiment to prove that circ‐100338 was indeed circRNA, as demonstrated that it was resistant to RNaseR digestion (Figure 1C).

### 3.2 Circ‐100338 functions as a sponge for miR‐141‐3p

**Figure 2:**
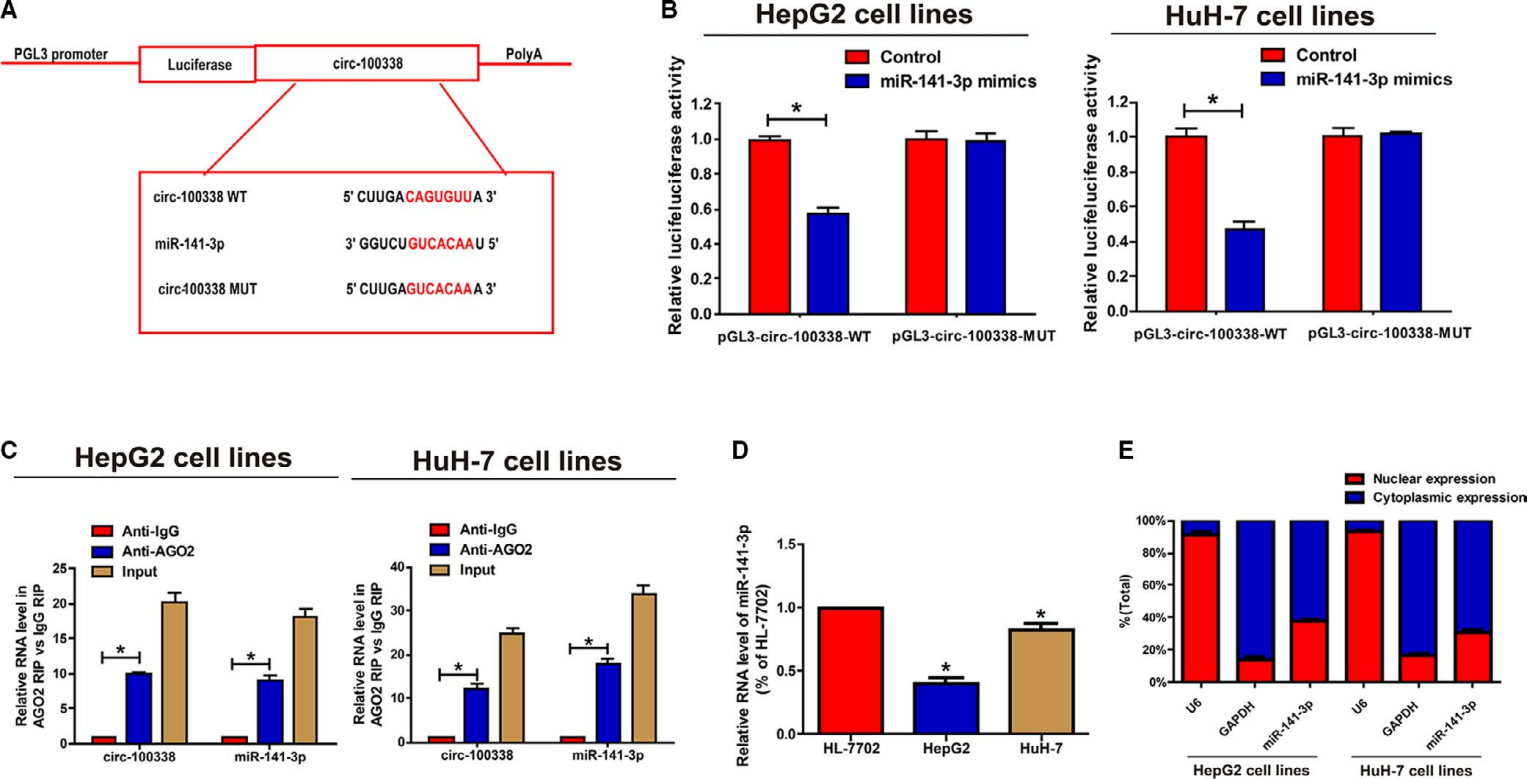
Bioinformatics analysis and experiments results manifested that circ‐100338 sponged miR‐141‐3p. A, Circ‐100338 WT and miR‐141‐3p binding sequence prediction and construction of the circ‐100338 MUT sequence. B, Dual luciferase reporter gene assay was performed to detect the binding relationship between miR‐141‐3p and circ‐100338 in HCC cells. C, RIP experiments showed that circ‐100338 and miR‐141‐3p were enriched in the product pulled down by Anti‐AGO2. D, The expression of miR‐141‐3p was significantly inhibited in HCC cell lines compared to HL‐7702 cell line. E, circ‐100338 was mainly located in the cytoplasm

Given the fact that circRNAs can act as a miRNAs sponge, the potential targets of circ‐100338 were predicted by bioinformatics. By crossing the predicted miRNAs, it was showed that miR‐141‐3p is the most likely complementary miRNA that binds to circ‐100338. To further investigate the relationships between predicted miRNAs and circ‐100338, we constructed a plasmid containing the mutant‐type or wild‐type circ‐100338 sequence (Figure 2A). The dual luciferase reporter assay was then used in HepG2 and HuH‐7, the relative luciferase activity in cells co‐transfected with circ‐100338 MUT and miR‐141‐3p mimics showed no significant difference with the control (Figure 2B). Subsequently, RIP assays were performed to verify that miR‐141‐3p directly targets circ‐100338 in HCC cells. The result pointed that circ‐100338 and miR‐141‐3p were specifically enriched in beads conjugated withAGO2 compared to control IgG immunoprecipitates (Figure 2C). We examined the levels of miR‐141‐3p in HCC cell lines and HL‐7702 cell line. QRT‐PCR results exhibited that the levels of miR‐141‐3p were obviously reduced in HCC cell lines compared to the HL‐7702 cell line (Figure 2D). To further investigate the specific mechanism of circ‐100338 involved in HCC, we measured the subcellular localization of circ‐100338 by nucleoplasmic separation experiments. Nuclear isolation experiments showed that circ‐100338 was mostly distributed in the cytoplasm of HCC cells (Figure 2E). This result again confirms that circ‐100338 may regulate HCC cells by posttranscriptional modification. In short, these results demonstrated that circ‐100338 targets miR‐141‐3p.

### 3.3 Circ‐100338 promotes the cell proliferation of HCC cells in vitro

**Figure 3:**
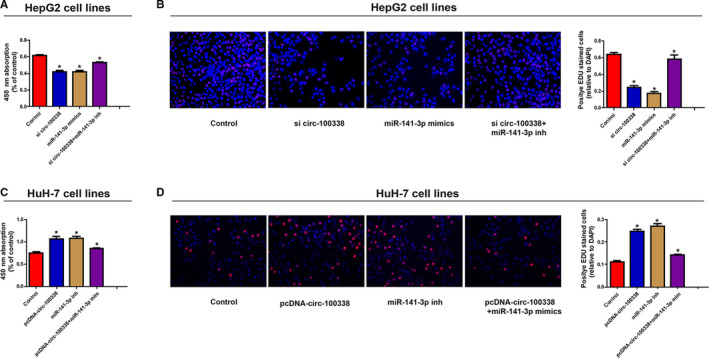
Circ‐100338 promotes proliferation of HCC cells in vitro. Knockdown of circ‐100338 inhibited HepG2 cell proliferation, and this inhibition can be reversed by transfection of miR‐141‐3p inhibitor. A, Absorbance at 450 nm measured by CCK8. B, Representative merge of the proliferating cells by the EDU assay. Overexpression of circ‐100338 promoted proliferation of HuH‐7 cells, and this inhibition can be reversed by transfection of miR‐141‐3p mimics. C, Absorbance at 450 nm measured by CCK8. D, Representative merge of the proliferating cells by the EdU assay

Previous studies showed that circ‐100338 is significantly increased in HCC cells, however, the effect of circ‐100338 on HCC cell function still needs further study. SiRNA against circ‐100338 and designed overexpress plasmid were used to silence and overexpress circ‐100338, respectively. The circ‐100338 siRNA and overexpression plasmid were transfected into HCC cells, respectively. CCK8 and EdU analysis showed that the proliferative ability of HepG2 cells was obviously downregulated by si‐circ‐100338, and this change could be reversed by miR‐141‐3p inhibitor (Figure 3A,B). Meanwhile, transfection with circ‐100338 overexpression vector significantly enhanced the proliferative capacity of HuH‐7 cells, and this enhancement was reversed by miR‐141‐3p mimics (Figure 3A,D). Collectively, these findings suggest that overexpression of circ‐100338 can stimulate the proliferative ability of HCC cells.

### 3.4 Circ‐100338 promotes HCC cell proliferation in vivo

**Figure 4:**
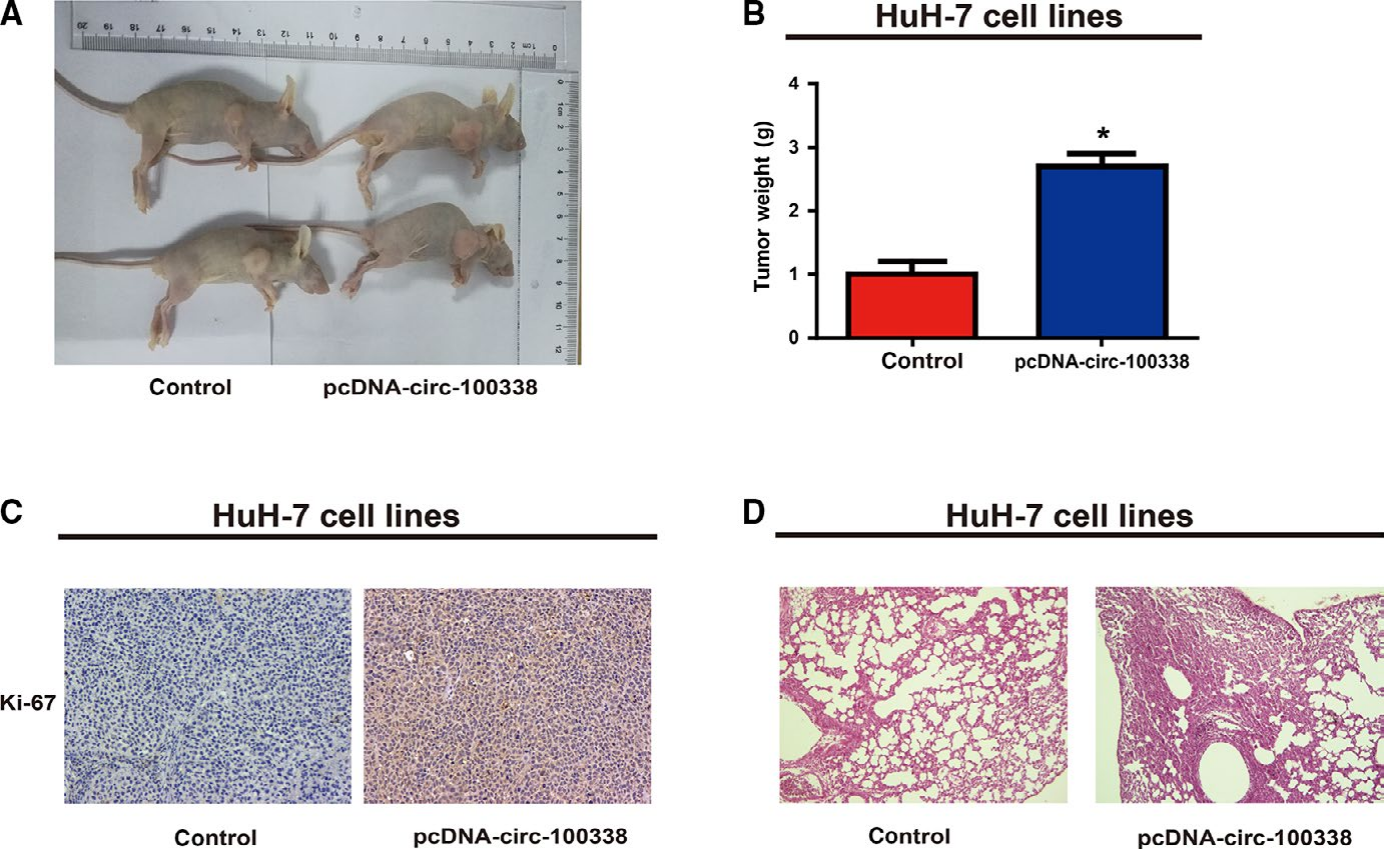
Circ‐100338 promotes HCC cell proliferation in vivo.(A) Representative images of xenografts tumor (two mice per group) in nude mice. (B)Tumor weight was monitored every 4 d for 28 d. (C) Representative images of IHC stained Ki‐67 are shown. (D) Representative images of HE stained Lung tissues are shown

To explore the function of circ‐100338 in HCC tumor growth, HuH‐7 cells transfected with blank or pcDNA‐circ‐100338 vector were subcutaneously injected into the flank of nude mice. The results indicated that overexpression of circ‐100338 increased the tumor volume (Figure 4A) and the tumor weight (Figure 4B) after the four‐week intratumorally injection. What's more, the result of immunohistochemistry showed that mice with circ‐100338 overexpression vector showed higher level of Ki‐67 (Figure 4C). Moreover results showed the destructions of lung tissues were more obvious in circ‐100338 vector group (Figure 4D).

### 3.5 Circ‐100338 regulates the target gene of miR‐141‐3p, ZEB1

**Figure 5:**
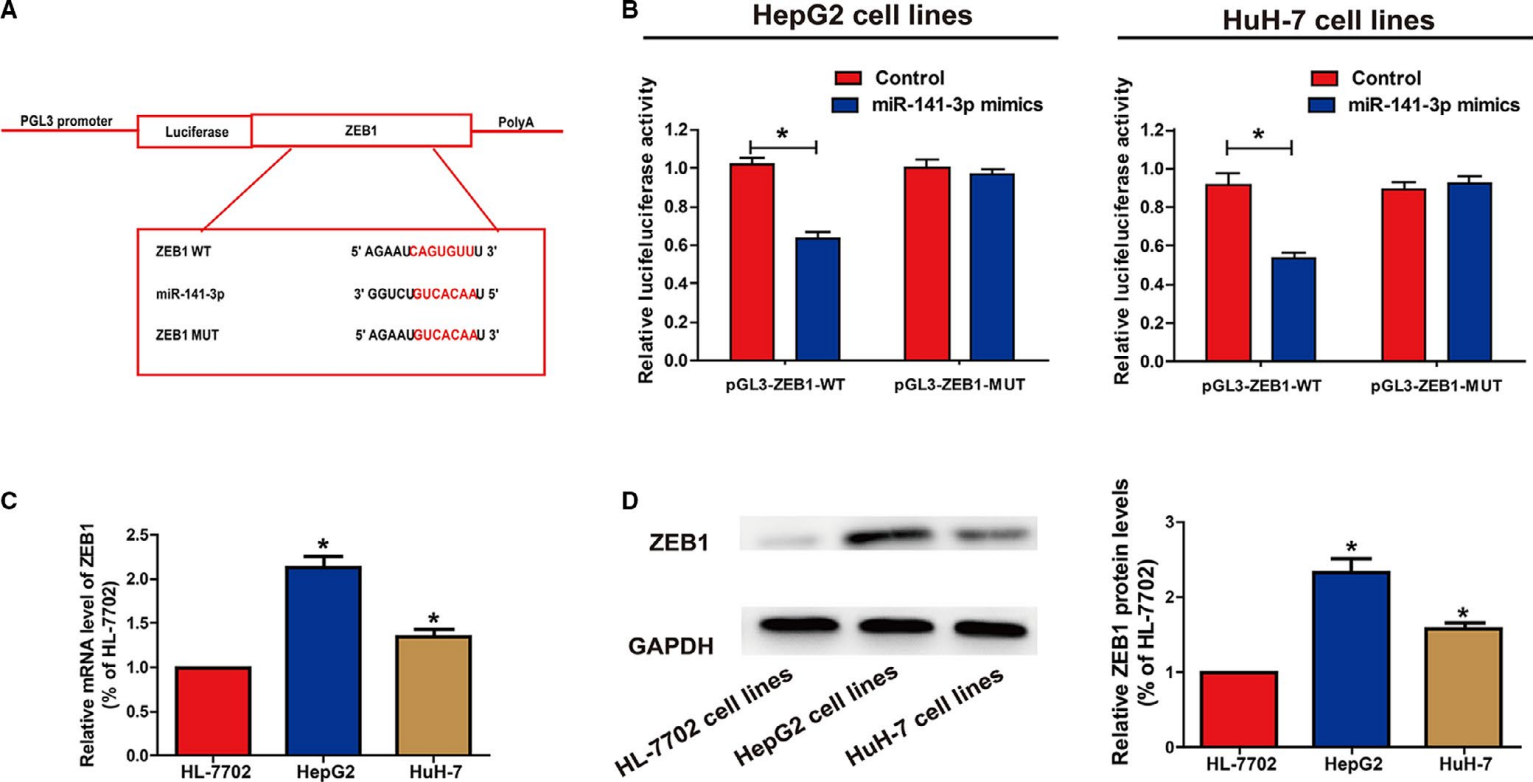
Circ‐100338 regulated ZEB1 by sequestering miR‐141‐3p. A, ZEB1 WT and miR‐141‐3p binding sequence prediction and construction of ZEB1 MUT sequence. B, The dual luciferase reporter gene showed that ZEB1 can bind to miR‐141‐3p. C, The expression of ZEB1 was significantly elevated in HCC cell lines compared to HL‐7702 cell line. D, Western blot analysis showed that the protein level of ZEB1 in the line was significantly upregulated in the G2 cell line and HuH‐7 cell compared to the normal liver cell lineHL‐7702

The target genes of miR‐141‐3p were predicted through bioinformatics analysis (Targetscan, DIANA, miRanda) and the intersections were taken. We found that the binding site of ZEB1 to miR‐141‐3p coincides with the binding site between circ‐100338 and miR‐141‐3p. To further confirm the relationship between miR‐141‐3p and ZEB1, we constructed the pGL3‐ZEB1‐WT and pGL3‐ZEB1‐MUT plasmids (Figure 5A). We then transfected HCC cells with the pGL3‐ZEB1‐WT and pGL3‐ZEB1‐MUT plasmids. Results showed that luciferase activity was significantly decreased in cells co‐transfected with pGL3‐ZEB1‐WT and miR‐141‐3p mimics compared to the control, luciferase activity was not altered in cells co‐transfected with pGL3‐ZEB1‐MUT and miR‐141‐3p mimics compared to the control (Figure 5B). We then examined the mRNA level of ZEB1 in HCC cell lines. Compared to the HL‐7702cell line, ZEB1 was obviously elevated in HCC cell lines (Figure 5C). Similarly, the protein levels of ZEB1 were also significantly increased in ACHN and A‐498 cell lines (Figure 5D). All together, these findings confirmed that ZEB1 is the target gene for miR‐141‐3p.

**Figure 6:**
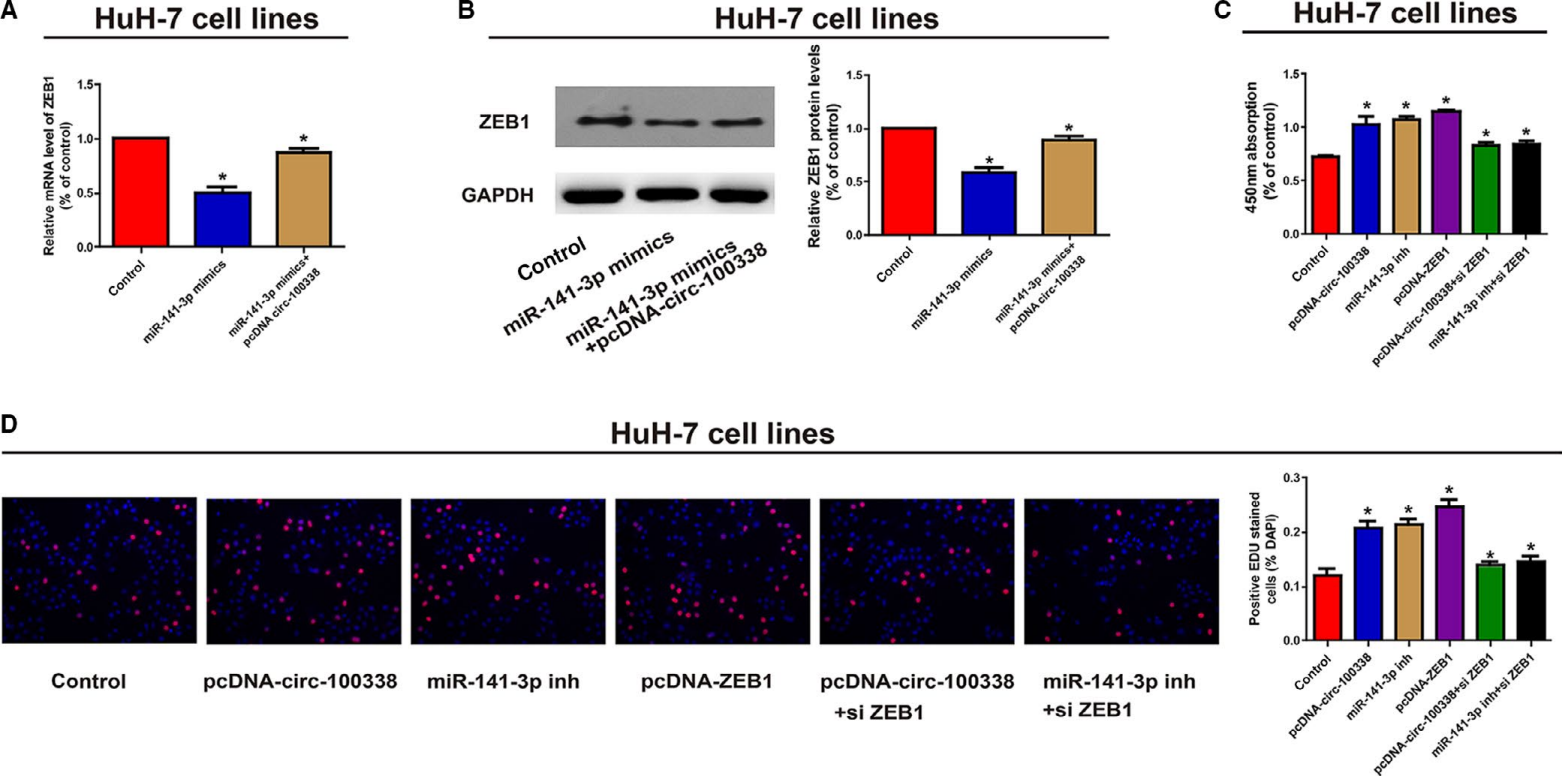
Circ‐100338 regulated cell proliferation via the circ‐100338/miR‐141‐3p/ZEB1 axis. A, HuH‐7 cells were transfected with miR‐141‐3p mimics with or without pcDNA‐circ‐100338 vector. The mRNA level of ZEB1 was measured. B, HuH‐7 cells were transfected with miR‐141‐3p mimicswith or without pcDNA‐circ‐100338 vector. The protein expression level of ZEB1 was examined, GAPDH was used as a control. C, The CCK8 assay was performed to determine the effect of circ‐100338/miR‐141‐3p/ZEB1 axis on the proliferation of HuH‐7 cells. D, An EdU assay was performed to determine the effect of circ‐100338/miR‐141‐3p/ZEB1 axis on the proliferation of HuH‐7 cells

After affirming that ZEB1 is a direct target of miR‐141‐3p, we were interesting as to whether or not circ‐100338 can regulate ZEB1 expression by binding to miR‐141‐3p. We transfected HuH‐7 cells with miR‐141‐3p mimics (Supplementary Figure 1B) and demonstrated that the ZEB1 protein and mRNA expression levels were obviously decreased, however, the addition of the circ‐100338 overexpression plasmid reversed the effects of miR‐141‐3p mimics (Figure 6A,B). Shortly, these data suggest circ‐100338 upregulated the levels of ZEB1 by sequestering miR‐141‐3p. QRT‐PCR analysis was carried out to evaluate the effects of transfection of pcDNA‐circ‐100338, miR‐141‐3p inhibitor and pcDNA‐ZEB1 on the proliferation of HCC cells. Compared with normal controls, the proliferation of HuH‐7 cells was significantly enhanced after transfection of pcDNA‐circ‐100338, miR‐141‐3p inhibitor, and pcDNA‐ZEB1, and this promotion was reversed by downregulating ZEB1 (Figure 6C,D). In a word, these experiments further validated the regulation of circ‐100338/ miR‐141‐3p/ ZEB1 pathway on HCC cell function.

## 4 DISCUSSION

HCC is one of the most common malignant tumor of the digestive system, and its occurrence is related to unrestricted proliferation of hepatocytes.^22^ Therefore, any cause of hepatocyte proliferation may lead to the occurrence of HCC. CircRNA has been shown to regulate cell migration or proliferation and can participate in the mechanism of HCC.^23^, ^24^ Studies have proved that piplartine can inhibit the proliferation of HCC cells,^21^ however, the specific mechanism is not clear. Therefore, we suspect that it is possible for piplartine to influence the proliferation of hepatocellular carcinoma and ultimately participate in the mechanism of hepatocellular carcinoma by regulating the expression of circRNA.

By reviewing the literature, we found that the study by Huang XY et al pointed out all circRNAs that may be involved in HCC. They identified 226 differentially expressed circRNAs using circRNA microarrays, of which 189 circRNAs were significantly upregulated and 37 were downregulated.^16^ The top six circRNAs that show differential levels in HCC are hsa_circRNA_100338, hsa_circRNA_102922, hsa_circRNA_104075,hsa_circRNA_101139, hsa_circRNA_102049 and hsa_circRNA_102533, respectively. Then we used piplartine to treat HCC cell lines to show that only circ‐100338 expression is significantly reduced compared with the control group. These results imply that piplartine may participate in the development of HCC by downregulating the expression of circ‐100338. Cell function experiments showed that si‐circ‐100338 significantly inhibited cell proliferation, indicating that circ‐100338 promotes HCC cell growth and plays an "oncogene" role. Therefore, a further study of the mechanism by which circ‐100338 facilitates HCC cell growth is of great importance for understanding how piplartine regulates the development, progression, and metastasis of HCC.

Through reporter experiments, RIP and other experiments, we determined that circ‐100338 acts as a sponge of miR‐141‐3p regulating the expression of its host gene ZEB1. In addition, cell function experiments confirmed that circ‐100338 may regulate HCC cell proliferation through the circ‐100338/miR‐141‐3p/ZEB1 axis. In summary, circ‐100338 plays as a ceRNA role in regulation of ZEB1 expression by sponging miR‐141‐3p in HCC, while piplartine can play a part in the progress of HCC by inhibiting the expression of circ‐100338.

Over recent years, miR‐141‐3p has been verified to suppress the growth and metastasis of tumor in multiple diseases, such as non‐small cell lung cancer,^25^ prostate cancer,^26^ colorectal cancer^27^ and hepatocellular carcinoma.^28^ Ye J and his colleagues find thatmiR‐141‐3p expression was substantially reduced in HCC cells and tissue samples.^29^ Hou X and his colleagues point out that miR‐141‐3p overexpression significantly decreased HCC cell proliferation, migration, and invasion by inhibiting epithelial‐mesenchymal transition (EMT).^30^ Zinc finger E‐box binding homeobox 1 (ZEBl) is one of the members of the ZEB family. ZEB1 contains two zinc finger structure clusters and one homologous domain, and the bilateral zinc finger clusters can bind to a specific sequence of DNA, thereby regulating transcription of the target gene.^31^ Studies have pointed out that ZEB1 can inhibit the levels of E‐cadherin, and paticipate in the metastasis and proliferation of various cancers.^32^, ^33^ Krebs AM et al found that the Zeb1, a EMT‐activator paticipates in cell metastasis and plasticity in pancreatic cancer.^34^ Accordingly, our study showed that upregulated circ‐100338 results in high levels of the miR‐141‐3p target gene ZEB1, which may cause excessive proliferation of HCC cells.

This research has several limitations. First, a large population‐based sample is required to further explore the clinical value of circ‐100338. Second, more target genes or miRNAs should be applied to interact with circ‐100338. Third, cell cycle assay should be performed to further explore the function of circ‐100338 in HCC. Fourth, the mechanism of circ‐100338 in hepatocellular carcinoma should be investigated via further assays. For example, whether circ‐100338 regulates the progression of hepatocellular carcinoma via the PI3K/AKT, NF‐kB, or Wnt pathway needs to be examined.

## 5 CONCLUSION

In summary, our research team showed that piplartine inhibits cell proliferation of hepatocellular carcinoma by circ‐100338 function as competitive endogenous RNA.

All data used in this study are available from the corresponding author upon reasonable request.

## CONFLICT OF INTEREST

None declared.

## AUTHORS’ CONTRIBUTIONS

XC and XY designed the concept and experiments. XC, PT and WZ performed the experiments. XC wrote the manuscript. XY revised the manuscript.

## Supporting information

Fig S1

Table S1

Supplementary Material

## Data Availability

All data are available upon request.
