# Supplementary figures and images for: Piplartine attenuates the proliferation of hepatocellular carcinoma cells via regulating hsa_circ_100338 expression
Source: Cancer Med. 2020 Apr 13;9(12):4265–73. doi: 10.1002/cam4.3043 (PMC7300402; doi:10.1002/cam4.3043)

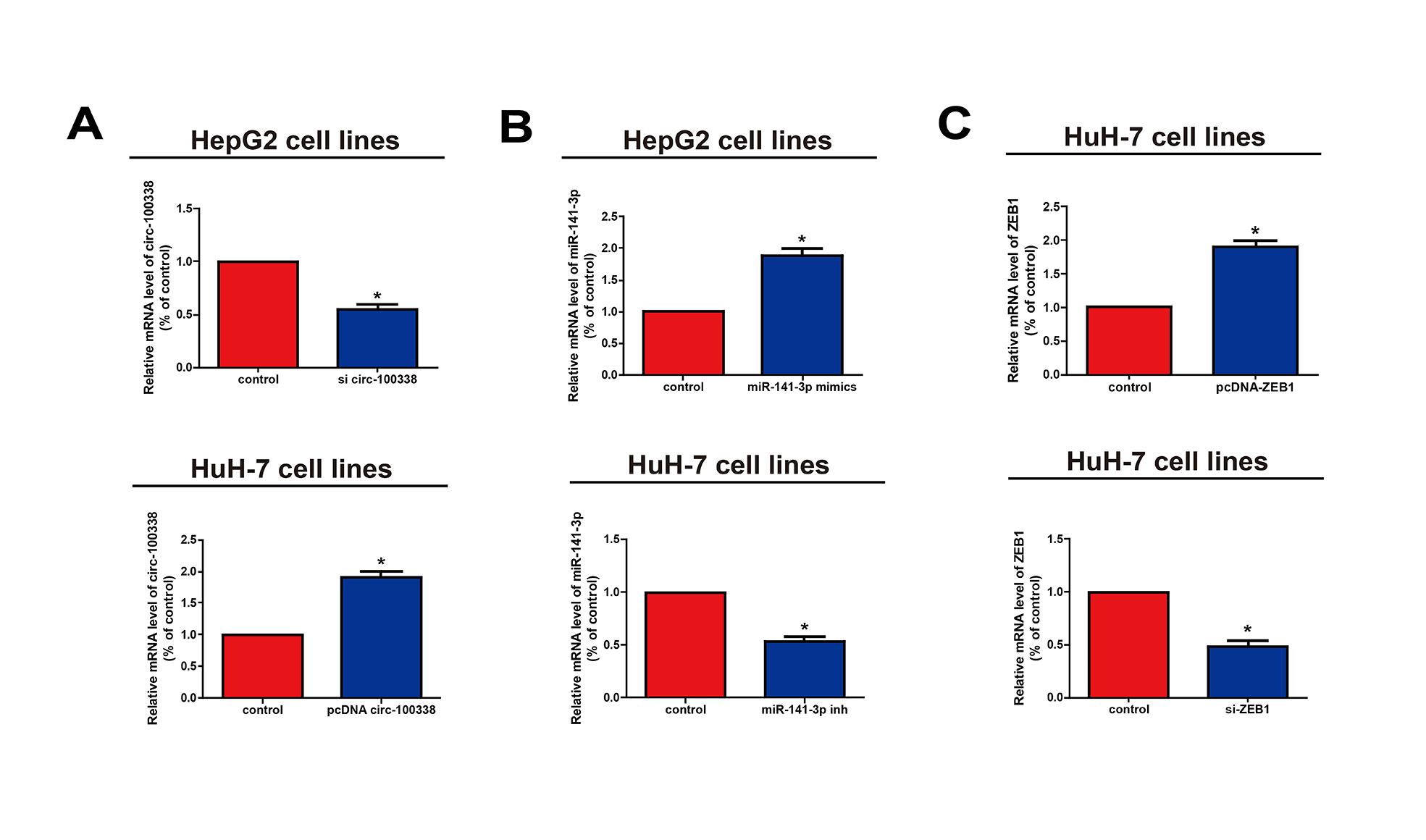

Supplement: Supplementary file 1 — Fig S1 [file CAM4-9-4265-s001.tif]
