# Supplementary material for: Piplartine attenuates the proliferation of hepatocellular carcinoma cells via regulating hsa_circ_100338 expression
Source: Cancer Med. 2020 Apr 13;9(12):4265–73. doi: 10.1002/cam4.3043 (PMC7300402; doi:10.1002/cam4.3043)
Supplement: Supplementary file 2 — Table S1 [file CAM4-9-4265-s002.doc]

| hsa_circRNA_100338 | F: AAAAGCAAGCAGTGCCCATA  R: GCTCGAATCAGGTCCACCA |
| --- | --- |
| hsa_circRNA_102922 | F: GCCTTCACCCTCCTTATCTCTA  R: TGGCATTCCATATTCAGCGA |
| hsa_circRNA_104075 | F: GAAGATGTCAAGCCCTTTAGC  R: GAGTTGCTTAGCTTTCATTTGTC |
| hsa_circRNA_101139 | F: CATCCGCTACCTCATCTCGT  R: GTTGCTACCACCACTCCCATA |
| hsa_circRNA_102049 | F: GAAGCATTTCATCAATAACCCTC  R: CAAAGCCACAGTCCATCACAG |
| hsa_circRNA_102533 | F: GCTGCCAAAAGCATAACCAA  R: GGATCTCGCTCCTGGAAGATG |
| GAPDH | F: GCACCGTCAAGGCTGAGAAC  R: CCCCTTTTCTGCTAAATGAACTCT |
| ZEB1 | F: GATGATGAATGCGAGTCAGATGC |
|  | R: ACAGCAGTGTCTTGTTGTTGT |
| U6 | F: CTCGCTTCGGCAGCACA |
|  | R: AACGCTTCACGAATTTGCGT |
| miR-141-3p | F: TCCCACCCAGTGCGATTTGTC |
|  | R: GTTGCTGGGAGGCTAAGATGAG |

**Supplementary Table 1. Primer sequence used for qRT‐PCR.**
