# Supplementary material for: Piplartine attenuates the proliferation of hepatocellular carcinoma cells via regulating hsa_circ_100338 expression
Source: Cancer Med. 2020 Apr 13;9(12):4265–73. doi: 10.1002/cam4.3043 (PMC7300402; doi:10.1002/cam4.3043)
Supplement: Supplementary file 3 — Supplementary Material [file CAM4-9-4265-s003.doc]

**Supplementary Figure 1. Cell transfection.** (A) SiRNA against circ-100338 and designed overexpress plasmid were used to silence and overexpress circ-100338 in HepG2 and HuH-7. (B) Mimics and inhibitor were used to overexpress and silence miR-141-3p in HepG2 and HuH-7. (C) PcDNA-ZEB1 and si-ZEB1 were used to and overexpress and silence ZEB1 in HepG2 and HuH-7.
